# Interaction of human dipeptidyl peptidase IV and human immunodeficiency virus type-1 transcription transactivator in *Sf9 *cells

**DOI:** 10.1186/1743-422X-7-267

**Published:** 2010-10-13

**Authors:** Felista L Tansi, Véronique Blanchard, Markus Berger, Rudolf Tauber, Werner Reutter, Hua Fan

**Affiliations:** 1 Institut für Biochemie und Molekularbiologie, Charité-Universitätsmedizin Berlin, Arnimallee 22 Berlin-Dahlem, Germany; 2 Zentralinstitut für Laboratoriumsmedizin und Pathobiochemie, Charité-Universitätsmedizin Berlin, Luisenstr. 7, 10117 Berlin, Germany

## Abstract

**Background:**

Dipeptidyl peptidase IV (DPPIV) also known as the T cell activation marker CD26 is a multifunctional protein which is involved in various biological processes. The association of human-DPPIV with components of the human immunodeficiency virus type-1 (HIV1) is well documented and raised some discussions. Several reports implicated the interaction of human-DPPIV with the HIV1 transcription transactivator protein (HIV1-Tat) and the inhibition of the dipeptidyl peptidase activity of DPPIV by the HIV1-Tat protein. Furthermore, enzyme kinetic data implied another binding site for the HIV1-Tat other than the active centre of DPPIV. However, the biological significance of this interaction of the HIV1-Tat protein and human-DPPIV has not been studied, yet. Therefore, we focused on the interaction of HIV1-Tat protein with DPPIV and investigated the subsequent biological consequences of this interaction in *Spodoptera frugiperda *cells, using the BAC-TO-BAC baculovirus system.

**Results:**

The HIV1-Tat protein (Tat-*BRU*) co-localized and co-immunoprecipitated with human-DPPIV protein, following co-expression in the baculovirus-driven *Sf9 *cell expression system. Furthermore, tyrosine phosphorylation of DPPIV protein was up-regulated in Tat/DPPIV-co-expressing cells after 72 h culturing and also in DPPIV-expressing *Sf9 *cells after application of purified recombinant Tat protein. As opposed to the expression of Tat alone, serine phosphorylation of the Tat protein was decreased when co-expressed with human-DPPIV protein.

**Conclusions:**

We show for the first time that human-DPPIV and HIV1-Tat co-immunoprecipitate. Furthermore, our findings indicate that the interaction of HIV1-Tat and human-DPPIV may be involved in signalling platforms that regulate the biological function of both human-DPPIV and HIV1-Tat.

## Background

Dipeptidyl peptidase IV (DPPIV, CD26, EC: 3.4.14.5) is a type II transmembrane sialoglycoprotein which belongs to the prolyl oligopeptidase family of serine proteases [1,2]. DPPIV cleaves dipeptides from the N-terminus of oligopeptides with proline or alanine in their penultimate position [3,4]. Physiological substrates of DPPIV include chemokines, peptide hormones and neuropeptides. However, additional roles have been assigned to DPPIV that are independent of its proteolytic activity. These include cell-adhesion by binding to collagen and fibronectin [5,6] as well as regulation of immune response by interacting with adenosine deaminase (ADA) and CD45 [7-9]. Thus, DPPIV serves as a receptor for the enzyme ADA, which in turn converts adenosine irreversibly to inosine, thereby preventing suppression of lymphocyte proliferation by adenosine.

There is evidence for the involvement of DPPIV in HIV-infection and the progression of AIDS-associated immune suppression, although DPPIV does not serve directly as a co-receptor of HIV infection [10] as was earlier postulated [11]. DPPIV is known to cleave many chemokines such as stromal cell derived factor 1 (SDF-1α/β), macrophage-derived chemokine (MDC) [12,13] and regulated on activation normal T cell expressed and secreted (RANTES) and regulate their biological functions. Intriguingly, cleavage of RANTES and SDF-1α, results in opposing effects regarding their anti-HIV activities. While truncation of RANTES by DPPIV increases its chemotactic activity via the C-C chemokine receptor 5 (CCR5) and thereby prevents HIV infection[14,15], cleavage of SDF-1α by DPPIV leads to reduced chemotactic activity and consequently promotes HIV infection via the C-X-C chemokine receptor 4 (CXCR4) [16]. The association of CXCR4 with DPPIV further supports the involvement of DPPIV in HIV infection [17]. Furthermore, it has been established that the HIV1 transactivator of transcription (HIV1-Tat) associates with and inhibits the enzymatic activity of DPPIV and thereby suppresses the co-stimulatory signalling of DPPIV [18-20]. The immunosuppressive effects of the HIV1-Tat protein seem to involve the interplay between HIV1-Tat protein, CXCR4 and DPPIV [21], since the HIV1-Tat protein is a known antagonist of CXCR4 [22].

The HIV1-Tat protein is a small 10-12 kDa protein that contains five distinct functional domains [23]. Its primary role is the transactivation of transcription of HIV proviral-DNA by binding to the transacting response element (TAR) on the proviral long terminal repeat (LTR) [24-26]. In the absence of Tat protein, the transcription of viral transcripts is low and results to production of shorter transcripts.

The HIV-Tat protein is secreted from HIV infected cells by a poorly studied mechanism and is suggested to have paracrine effects on uninfected cells of HIV infected patients [27]. Extracellular Tat protein re-enters cells via lipid rafts and caveolar up-take [28] by interacting with cell surface receptors such as heparan sulphate proteoglycans [29], the integrin receptors α5β1 and αvβ3 and the extracellular matrix proteins fibronectin and vitronectin [30,31]. The subsequent outcome of such re-entry of Tat into cells is diverse and poorly studied. Studies with recombinant Tat protein reveal that extracellular Tat taken-up by cells, translocates to the nucleus where it modulates amongst others, the expression of a wide range of genes such as cytokines [32,33], chemokine receptors [34] and major histocompatibility complex (MHC) [35,36]. Post-translational modifications of the Tat protein are well documented [37-39], which offer the protein a wide range of functions that are still poorly understood.

The association of extracellular HIV1-Tat protein to DPPIV on the cell membrane and its internalization due to this association is unresolved [18,40]. These discrepancies may lie in the methods used and also the observation that extracellular HIV1-Tat protein is an opportunist that uses different cell surface proteins to attach to the surfaces of different cell types prior to its internalization. Thus, purified HIV1-Tat protein can be internalized by different cell types irrespective of the expression of DPPIV or some of the already characterised cell surface proteins it uses for internalization [28-30]. Presently, it is known that Tat protein plays a vital role in the pathogenesis of AIDS. However, the multifaceted nature of Tat protein indicates that many of its characteristic roles in HIV-infection and the progression of AIDS are still unknown. Inhibition of DPPIV enzyme activity and suppression of DPPIV-dependent T-cell growth [41], are known characteristics of the Tat protein as well as its interaction with DPPIV. However, the mechanism behind the Tat protein-mediated suppression of DPPIV-dependent T-cell growth has not been elucidated yet. Whether the inhibition of the enzymatic activity of DPPIV by HIV1-Tat protein is the sole mediator of this effect is also not known.

Therefore, we developed a simple strategy to study the effects of HIV1-Tat protein on human-DPPIV and vice versa in *Sf9 *cells using the BAC-TO-BAC baculovirus expression system. Co-expression of HIV1-Tat and human-DPPIV in *Sf9 *cells offered relatively high yields of the proteins within a very short time. We show that HIV1-Tat protein co-associates with human-DPPIV in *Sf9 *cells. Furthermore, we demonstrate that tyrosine phosphorylation of DPPIV is induced by purified recombinant Tat protein, whereas serine-phosphorylation of HIV1-Tat is reduced due to its co-expression with human-DPPIV.

## Results and Discussion

### Human-DPPIV and HIV1-Tat co-associate in co-infected *Sf9 *cells

To verify whether HIV1-Tat associates with human-DPPIV, *Sf9 *cells co-infected with Tat and DPPIV recombinant baculoviruses were harvested 48 h and 72 h post infection and stained with antibodies as described under methods. Images of the stained cells were made at a 100-fold magnification by confocal microscopy.

**Figure 1:**
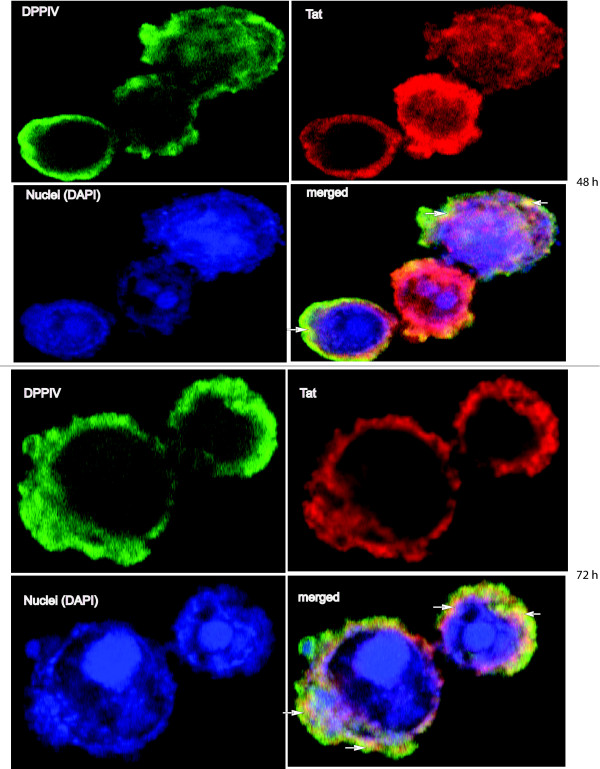
**Co-localization of human-DPPIV and HIV1-Tat in *Sf9 *cells**. *Sf9 *cells co-infected with Tat and DPPIV recombinant baculoviruses were harvested at the indicated time then fixed on culture slides and double-stained with the antibody combination: anti-Tat mAb/anti-mouse-Cy3 (*red*) and anti-DPPIV pAb/anti-rabbit-FITC (*green*). Nuclei were stained with DAPI (*blue*) and images were made at 100-fold magnification by confocal laser scanning microscopy. Co-localization of HIV1-Tat and human-DPPIV protein takes place at the cell membrane of merged images (*white arrows*).

**Figure 2:**
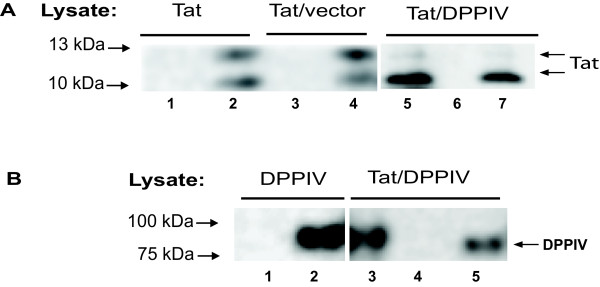
**Co-immunoprecipitation of human-DPPIV and HIV1-Tat protein from *Sf9 *cells**. Single expression or co-expression of Tat with DPPIV was performed for 72 hours, afterwards the cells were solubilised and lysates subjected to immunoprecipitation, (IP). (**A**) IP was conducted with anti-Tat mAb (lanes 2, 4 and 5) or with anti-DPPIV pAb (lanes 1, 3 and 7). Lane 6 was control conducted with protein-A-sepharose without antibody. Tat/vector represents control cells co-infected with Tat and a pFASTBAC1 vector virus. Each immunoprecipitate was separated by SDS-PAGE and the gels blotted and probed for with anti-Tat mAb. (**B**) IP was conducted with anti-Tat mAb (lanes 1 and 5) or with anti-DPPIV pAb (lanes 2 and 3) or with protein-A-sepharose without antibody (lane 4). The immunoprecipitates were subsequently blotted and probed for with anti-DPPIV pAb. The figure represents one of three independent experimental results.

Confocal microscopy detected the Tat protein predominantly in the nucleus and cytosol and to a lesser extent on the cell membrane (Figure 1). As expected, the DPPIV protein is predominantly located on the cell membrane. Staining the nuclei of cells with DAPI made it easy to distinguish the nuclei from other compartments. HIV1-Tat and human-DPPIV co-localize at the cell membrane of *Sf9 *cells as indicated with arrows on merged images (Figure 1, white arrows). Controls conducted with single and co-infected *Sf9 *cells completely ruled-out non-specific binding of the antibodies used (see Additional File 1: Negative controls for confocal microscopy). We further confirmed the association of HIV1-Tat with human-DPPIV using co-immunoprecipitation tests. Implementing antibodies against Tat or DPPIV, both DPPIV and Tat protein could be co-immunoprecipitated from *Sf9 *cells after 72 h co-expression (Figure 2A and 2B). In control samples of single expressions, neither unspecific precipitation of Tat by the anti-DPPIV pAb (Figure 2A, lane 1) nor of DPPIV by the anti-Tat mAb was seen (Figure 2B, lane 1). Furthermore, unspecific binding of the proteins to the protein-A-sepharose used was not detected, implying that Tat and DPPIV undergo binding which initiates their co-immunoprecipitation from co-infected cells. Immunoprecipitation of Tat with anti-Tat mAb always yielded a double band at 10 kDa and 13 kDa. We noticed that the 13 kDa band had a higher intensity than the 10 kDa protein band when Tat was expressed alone (Figure 2A, lane 2). Interestingly, when Tat was co-expressed with DPPIV, immunoprecipitation with Tat or DPPIV specific antibodies yielded predominantly the 10 kDa band (Figure 2A, lanes 5 and 7). Co-expressing Tat and a control recombinant baculovirus prepared by transfecting the pFASTBAC1 cloning vector bacmid in *Sf9 *cells did not lead to a difference in the intensities of the 10 kDa and 13 kDa protein bands (Figure 2A, lane 4). This suggests that the HIV1-Tat protein due to co-expression with human-DPPIV, but not the high virus titre used is predominantly in the 10 kDa form. Interestingly, co-immunoprecipitation of Tat and DPPIV was only possible when whole-cell lysates were used. Using crude membrane extracts or mixed cell lysates of independently expressed human-DPPIV and HIV1-Tat protein yielded no binding of the proteins in immunoprecipitation tests. Furthermore, solubilisation of Tat/DPPIV co-expressing *Sf9 *cells with Triton X100 which destroys protein-protein contacts completely abolished their co-immunoprecipitation. Taken together, it is likely that binding of Tat and DPPIV requires some modifications or factors which are only disposed when both proteins are co-expressed.

### Serine-phosphorylation of HIV1-Tat protein is reduced due to human-DPPIV

**Figure 3:**
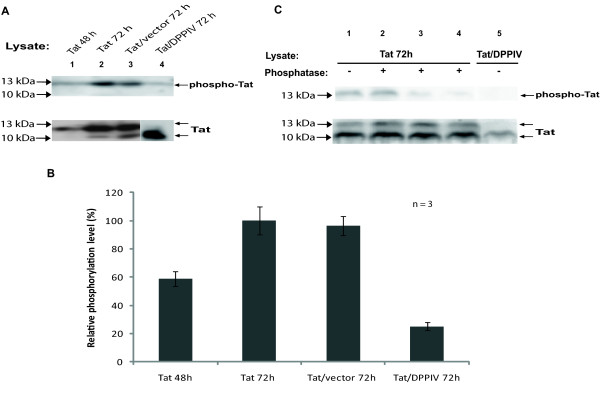
**Human-DPPIV causes a decrease in phosphorylation of HIV1-Tat in *Sf9 *cells**. (**A**) Lysates of Tat-expressing *Sf9 *cells harvested at the indicated time were subjected to IP with anti-phospho-serine mAb (upper panel) or anti-Tat mAb (lower panel). Each immunoprecipitate was separated by SDS-PAGE, blotted and subsequently probed for with anti-Tat mAb. The band in lane 4 (lower panel) was cut from another position of the same blot. (**B**) The intensities of phospho-Tat protein bands were determined with the QuantityOne software and calibrated against the respective intensities of the total proteins. Tat being expressed alone for 72 h was taken as standard (= 100%). Each bar represents the relative phosphorylation level deduced from 3 independent experiments ± standard deviations as a percentage of the standard. (**C**) Lysates of Tat-expressing *Sf9 *cells were treated with different concentrations of alkaline phosphatase for 2 h at 37°C as follows: 1 U alkaline phosphatase/500 μg protein (lane 2), 1 U alkaline phosphatase/200 μg protein (lane 3) or 1 U alkaline phosphatase/100 μg protein (lane 4). Control samples were left untreated (lane 1 and lane 5) then immunoprecipitated with the anti-phospho-serine mAb (upper panel) or anti-Tat mAb (lower panel). The immunoprecipitates were blotted and subsequently probed for with anti-Tat mAb.

To confirm whether the double Tat protein bands resulted from posttranslational modifications, we verified if Tat expressed in the *Sf9 *cell system was phosphorylated. Precipitation of Tat protein from Tat- and Tat/DPPIV-expressing *Sf9 *cells with anti-phospho-serine mAb was achieved (Figure 3A, upper panel). Only the 13 kDa band, but not the 10 kDa band was precipitated by the anti-phospho-serine mAb, opposed to both 10 kDa and 13 kDa bands precipitated by the anti-Tat mAb from the same lysates (Figure 3A, lower panel). Furthermore, only a faint 13 kDa band was precipitated from Tat/DPPIV-expressing *Sf9 *cells, suggesting that phosphorylation of HIV1-Tat protein was reduced due to its co-expression with DPPIV. Assuming that the level of phosphorylation of Tat protein being expressed alone for 72 h is 100%, the phosphorylation level after 48 h was 59% (Figure 3B). Only 22% of phospho-Tat protein was realized after 72 h co-expression with DPPIV. Co-infecting *Sf9 *cells with HIV1-Tat- and the control vector-virus did not influence the level of serine-phosphorylation of Tat, suggesting that the decrease in the pool of phospho-Tat in Tat/DPPIV samples was not due to the high titre of virus used in co-infection, but rather due to DPPIV.

To further confirm whether the 13 kDa band was precipitated due to phosphorylation and not unspecific binding to the antibody, lysates of HIV1-Tat being expressed alone or together with human-DPPIV for 72 h were either treated with alkaline phosphatase or left untreated, then subsequently immunoprecipitated with the anti-phospho-serine mAb or anti-Tat mAb. The treatment of lysates with increasing concentrations of alkaline phosphatase led to a decrease in the immunoprecipitation of phospho-Tat protein with anti-phospho-serine mAb (Figure 3C, lanes 2-4, upper panel), so that treatment with 1 U alkaline phosphatase/100 μg proteins removed almost all the phosphates and almost completely abolished precipitation of Tat by the anti-phospho-serine mAb (Figure 3C, lane 4, upper panel). Opposed to this, immunoprecipitation with anti-Tat mAb yielded relatively uniform intensities of the Tat protein, irrespective of the concentration of the alkaline phosphatase used (Figure 3C, lanes 2-4, lower panel). The HIV1-Tat protein (Tat-BRU) expressed in this study has only one tyrosine residue (Y-47). Precipitation of Tat protein with anti-phospho-tyrosine mAb was not detected.

It has been established earlier, that phosphorylation of Tat protein takes place on serine-16 and serine-46 and is important in regulating the level of transcription of HIV1 by Tat protein. Mutation of both serine-16 and serine-46 to alanine resulted in a 3-fold decrease in Tat transactivation activity [38]. Our results show that serine phosphorylation of the HIV1-Tat protein is reduced due to its co-expression with DPPIV, suggesting that DPPIV plays a regulatory role in HIV transcription. Besides phosphorylation, other post-translational modifications of HIV1-Tat protein such as acetylation, lysine-methylation and ubiquitination contribute in regulating Tat's function as a transcription activator [37,42,43]. Although we did not investigate the effect of DPPIV on the acetylation, methylation or ubiquitination of Tat protein in *Sf9 *cells, it seems likely that some of these modifications are altered when HIV1-Tat is co-expressed with human-DPPIV. Tat being expressed alone yields both a 10 kDa and a 13 kDa modified protein, but the 10 kDa protein predominantly when co-expressed with DPPIV. Phosphorylation alone (on serine-16 and serine-46) cannot result to the 3 kDa molecular mass difference observed in the Tat protein bands. Furthermore, treatment of lysates of Tat-expressing *Sf9 *cells with increasing concentrations of alkaline phosphatase did not result in a significant increase or decrease in the intensity and mass of the Tat protein bands precipitated with anti-Tat mAb (Figure 3C, lower panel), but significantly reduced the precipitation of phospho-Tat by anti-phospho-serine mAb (Figure 3C, upper panel). These findings suggest that phosphorylation and probably other unknown posttranslational modifications of HIV1-Tat are altered due to its co-expression with human-DPPIV, which are involved in regulating Tat transactivation activity.

### HIV1-Tat induces tyrosine phosphorylation of human-DPPIV in *Sf9 *cells

**Figure 4:**
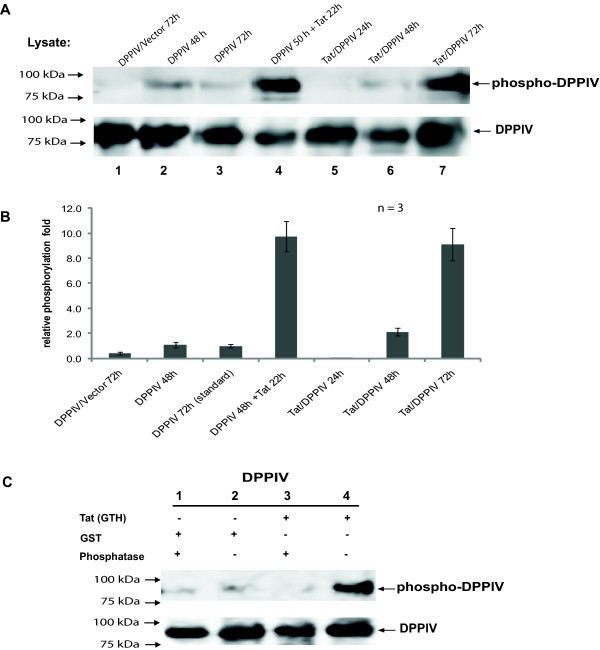
**HIV1-Tat induces tyrosine phosphorylation of human-DPPIV in *Sf9 *cells**. (**A**) *Sf9 *cells were infected with the indicated recombinant baculoviruses. After 50 h a portion of the DPPIV-expressing cells were treated with 2.7 nM final concentration of purified recombinant Tat protein (GTH: GST-Tat-His), and cultured for further 22 h. The resulting cell lysates were subsequently subjected to IP with anti-phospho-tyrosine mAb (upper panel) or anti-DPPIV pAb (lower panel). Each immunoprecipitate was separated by SDS-PAGE, blotted and probed for with anti-DPPIV pAb. DPPIV/vector: cells co-infected with DPPIV- and a control pFASTBAC1-vector recombinant virus. (**B**) The band intensities of phospho-DPPIV protein (in B, upper panel) were quantified with the QuantityOne software (Bio-Rad) and calibrated against the total DPPIV protein. The value of DPPIV being expressed alone for 72 h was set as standard (= 1) and the relative phosphorylation fold deduced as quotients of the values got for the other samples and the standard. (**C**) *Sf9 *cells were infected with DPPIV recombinant baculovirus and cultured for 68 h then treated with GST-Tat-His or GST and cultured for further 4 h. After solubilisation, each lysate was divided into two equal portions and either treated with alkaline phosphatase (lanes 1 and 3) or left untreated (lanes 2 and 4). The lysates were subjected to IP with anti-phospho-tyrosine mAb (upper panel) or anti-DPPIV pAb (lower panel). Immunoprecipitates were separated by SDS-PAGE, blotted and subsequently probed for with anti-DPPIV pAb. The figure represents one of three independent experimental results.

DPPIV expressed in *Sf9 *cells was phosphorylated on tyrosine residues and could be immunoprecipitated with the anti-phospho-tyrosine mAb. The level of phosphorylation was increased in the lysates of DPPIV-expressing cells that were treated with 2.7 nM final concentration of the purified recombinant Tat protein (GST-Tat-His) for 22 h and also in Tat/DPPIV-expressing cells after 72 h co-expression (Figure 4A, lanes 4 and 7 upper panel). With reference to untreated DPPIV being expressed alone for 72 h (Figure 4A, lane 3), the phosphorylation of DPPIV after application of purified Tat protein was increased by 9 folds and that of Tat/DPPIV sample by 8 folds (Figure 4B). Co-infecting cells with DPPIV and the control vector virus for 72 h did not lead to the increase in the pool of phospho-DPPIV seen in Tat/DPPIV-expressing cells (Figure 4A, lanes 1 and 7), making it evident that HIV1-Tat and not the high titre of virus used in co-expressions is responsible for the induction of DPPIV tyrosine phosphorylation.

It has been established, that the N-terminal sequence of HIV1-Tat protein is responsible for the inhibition of the enzymatic activity of DPPIV [44]. The purified recombinant Tat protein applied to cells in this study carried an N-terminal Glutathione-S-Transferase (GST) fusion-tag which eased its purification from *E. coli*. The GST-Tat-His protein revealed a strong ability to transactivate the HIV1-LTR promoter in a stably transfected HeLa cell line (see Additional File 2: Determination of the transactivation activity of recombinant Tat protein), but was unable to inhibit the enzymatic activity of purified human-DPPIV *in vitro *(see Additional File 3: Determination of the ability of recombinant Tat protein to inhibit the enzymatic activity of DPPIV), due to the N-terminal GST-tag. To proof that Tat itself and not the long GST-tag on the recombinant GST-Tat-His protein induced tyrosine phosphorylation of DPPIV, *Sf9 *cells were infected with DPPIV recombinant baculovirus for 68 h, then a portion of the cells subsequently treated with the purified recombinant GST-Tat-His or GST protein for 4 h. Reducing the induction time to 4 h still led to a significant increase in tyrosine phosphorylation of DPPIV in the Tat-treated samples (Figure 4C, lane 4), opposed to the GST-treated sample (Figure 4C, lanes 1 and 2), indicating that the recombinant GST-Tat-His protein induced tyrosine phosphorylation of human-DPPIV in *Sf9 *cells due to Tat and not the GST-tag. Furthermore, treatment of sample lysates with alkaline phosphatase prior to immunoprecipitation with the anti-phospho-tyrosine mAb abolished the immunoprecipitation of DPPIV (Figure 4C, lane 3), indicating that the anti-phospho-tyrosine mAb used is specific for recognition of tyrosine-phosphorylated proteins.

Co-expression of Tat and DPPIV yielded only traces of phospho-DPPIV at expression durations 24 h and 48 h. At 72 h co-expression, the pool of phospho-DPPIV increased significantly (Figure 4A, lane 5-7), suggesting that the level of the Tat protein expressed at this stage may play a role in the induction of tyrosine phosphorylation of DPPIV. However, detectable traces of Tat protein were secreted to the culture medium at this stage of expression (result not shown) suggesting that the concentration of Tat protein in the culture medium may also play a role in tyrosine phosphorylation of DPPIV. Selective activation of a variety of protein kinases by extracellular Tat protein in other cell systems has been documented [45-47].

Taken together, our results using the purified GST-Tat-His protein suggest that the inhibition effect of Tat protein on the enzyme activity of DPPIV is not a prerequisite for its induction of the phosphorylation of DPPIV on tyrosine residues. Furthermore, treating DPPIV- and Tat/DPPIV-expressing *Sf9 *cells previously cultured for 48 h with the DPPIV-inhibitor Ile-Pro-Ile for 4.5 h did not induce an increase in tyrosine phosphorylation of DPPIV in the cells (result not shown). These observations suggest that N-terminal binding of HIV1-Tat protein to the active site of human-DPPIV may result in inhibition, whereas interaction of Tat protein to the glycan moieties may lead to phosphorylation of DPPIV [19] and result to different signalling cascades.

Interestingly, DPPIV-inhibitor dependent induction of tyrosine phosphorylation of several proteins in resting T cells was reported earlier [48]. However, high expression of DPPIV is restricted to activated T cells [49] which explains why the study did not detect phospho-DPPIV. Furthermore, Tat-dependent induction of apoptosis in resting T cells was also documented which was associated with enhanced activation of cyclin-dependent kinases [50]. Generally, apoptotic effects of specific inhibitors of DPPIV have not been reported so far, which makes them suitable therapeutic drugs in the treatment of diabetes mellitus type-2 [51]. Taken together, these findings support our observation that the effect of Tat protein on DPPIV is not only dependent on its inhibition capacity, but also dependent on the activation of other signalling cascades involving DPPIV tyrosine phosphorylation. Recently, insulin-dependent tyrosine phosphorylation of DPPIV in rat liver was reported and the DPPIV protein levels were shown to be down-regulated by 40% in both plasma membrane and Golgi/ER fractions after addition of the tyrosine phosphatase inhibitor bpV (phen) [52]. This finding in rat liver revealed that tyrosine phosphorylation of DPPIV is associated with the stability and signalling activity of DPPIV. The stability and level of DPPIV is important in its interaction with CD45 and in the co-stimulation of T cell activation. In accordance with this, high level of DPPIV expression is restricted to activated T cells [49].

The simple strategy designed in the underlying work using the BAC-TO-BAC baculovirus-driven *Sf9 *expression system allows co-expression of high yields of both HIV1-Tat and human-DPPIV and is suitable for the investigation of their interaction in the cells. Owing to the high expression levels, protein-protein associations whose detection is limited in other expression systems by low expression levels was possible. Our findings in the *Sf9 *insect cell system can be argued by others as being an artefact. However, this can be ruled out, since both HIV1-Tat and human-DPPIV proteins purified from the same cell system retain their specific activities. Furthermore, the crystal structure of human-DPPIV protein expressed and purified from this cell system did not reveal any differences to that purified from animal tissues [53].

Shortly before this work was submitted for publication, the crystal structure of HIV1-Tat protein in complex with human p-TEFb was elucidated following their co-expression by the same strategy and in the same cell system used in the underlying work [54]. This supports the fact that the cell system is suitable for the expression of the HIV1-Tat protein with binding partners of human origin and strengthens the fact that the protein modifications in our research is due to the binding partners used and not due to the cell system.

## Conclusion

This work points out the significance of the interaction of HIV1-Tat and human-DPPIV and reveals further insights towards understanding HIV infection and the progression of AIDS. It demonstrates the co-localization and binding of HIV1-Tat protein and human-DPPIV in *Sf9 *cells. Furthermore, it reveals that serine-phosphorylation of Tat is significantly reduced due to its co-expression with human-DPPIV, whereas tyrosine phosphorylation of DPPIV is strongly induced by the HIV1-Tat protein. The findings further indicate that the inhibition effect of Tat on the enzymatic activity of human-DPPIV is not a prerequisite for its induction of the tyrosine phosphorylation of DPPIV.

## Materials and methods

### Construction and expression of human-DPPIV and HIV1-Tat in *Sf9 *cells

Expression and purification of enzymatically active DPPIV from insect cells was reported earlier [55]. Full length HIV1-Tat cDNA, pre-cloned into the mammalian vector pC63.4.1, (NIBSC, AIDS Research and Reference Reagent Program) was amplified by PCR and ligated in the pCR-Blunt vector (Invitrogen). The cDNA was then sub-cloned into the EcoR1 and Xba1 sites of pFASTBAC1 vector. After verification of its sequence by the Sanger dideoxy chain termination method [56], the Tat-pFASTBAC1 plasmid was transformed in the *E. coli *DH10Bac cells (Invitrogen). Preparation of recombinant bacmid, transfection into the baculovirus-driven *Sf9 *cells and production of recombinant baculovirus was done according to the manufacturer's instructions. For protein expression purposes, *Sf9 *cells at a density 2 × 10^6 ^cells/ml serum-deficient medium were infected with the recombinant baculovirus at the multiplicity of infection (MOI) = 1. For co-expression of HIV1-Tat and human-DPPIV, cells at the given density were co-infected with both HIV1-Tat- and DPPIV-recombinant baculoviruses at the MOI ratio 1:1.

### Antibodies

The antibodies used were anti-Tat mAb (NIBSC, AIDS Research and Reference Reagent Program), rabbit-anti-human-DPPIV pAb (anti-DPPIV pAb, 9/9 prepared in our laboratory), Cy3-conjugated-anti-mouse antibody (anti-mouse-Cy3, Sigma), FITC-conjugated-anti-rabbit antibody (anti-rabbit-FITC, Sigma), anti-phospho-serine mAb (Sigma, P3430 clone PSR-45) and anti-phospho-tyrosine mAb (Sigma, P3300 clone PT-66).

### Immuno-staining and confocal microscopy

*Sf9 *cells co-infected with the Tat- and DPPIV-recombinant baculoviruses were harvested 48 and 72 h post infection and assessed. The cells were washed, fixed on culture slides and permeabilized with 0.1% Triton X100 in PBS for 10 min then blocked with 1% BSA and 1% non-fat milk in PBS, pH 8.0. The cells were incubated with the anti-Tat mAb (diluted 1:200 in PBS containing 0.5% BSA) for 6 h, then washed twice with PBS and incubated with the anti-DPPIV pAb (diluted 1:200 in PBS containing 0.5% BSA) overnight. After washing 3 times with PBS the secondary antibodies anti-mouse-Cy3 (diluted 1:500 in PBS containing 0.5% BSA) and anti-rabbit-FITC (diluted 1:200 in PBS containing 0.5% BSA) were added to the cells simultaneously and incubated in the dark for 30 min. The cells were washed 3 times with PBS then the nuclei stained by incubating the cells for 10 min in 10 μg/ml 4' 6-diamidin-2-phenylindole dihydrochloride (DAPI) in PBS containing 3% formaldehyde. The cells were then washed 3 times with PBS, air-dried, immersed in mounting solution and assessed by confocal laser scanning microscopy (Carl Zeiss LSM 410) and images made at a 100-fold magnification. Control experiments were performed as follows: (1) *Sf9 *cells expressing either only Tat or DPPIV were double-stained and assessed under the same conditions like the Tat/DPPIV-expressing cells. (2) Tat/DPPIV-expressing *Sf9 *cells were stained with a combination of the antibodies anti-Tat mAb/anti-mouse-Cy3/anti-rabbit-FITC or with the combination anti-DPPIV pAb/anti-rabbit-FITC/anti-mouse-Cy3. The cells were then assessed by confocal microscopy as mentioned above.

### Cell lysis and solubilisation of protein

Infected *Sf9 *cells were harvested at the indicated expression duration and washed once with PBS, then resuspended and sonicated in solubilisation buffer (PBS containing 2% n-dodecyl-β-D maltoside, 500 KU Trasylol and 0.5 mM DTT). Solubilisation was performed by 4 h incubation at 4°C with head over tail rotation. Soluble proteins were fractionated by centrifugation at 18,000 rpm (29,703 × g) for 30 min.

### Determination of protein concentration

Protein concentrations were determined using the BCA Protein Assay Reagent Kit (Pierce).

### Dephosphorylation of proteins in lysates

In order to remove phosphate groups from the residues of proteins in lysates, 500 μl samples (800 μg total protein) were diluted with 400 μl distilled water and 100 μl 10 × dephosphorylation buffer (Fermentas). A mixture of shrimp- and calf alkaline phosphatase were added to the final concentrations 1 U/100 μg protein, 1 U/200 μg and 1 U/500 μg protein. The reaction mixtures were subsequently incubated for 2 h at 37°C.

### Immunoprecipitation

The following antibodies were used: anti-Tat mAb, anti-DPPIV pAb, anti-phospho-serine mAb and anti-phospho-tyrosine mAb. For each immunoprecipitation test, 2 μg of the respective antibodies were diluted with 1 ml PBS, pH 8.0 and immobilized on 10 mg protein-A/G-Sepharose (Pierce) by rocking overnight at 4°C. Unbound antibody was removed by washing twice with 1 ml PBS. Unless otherwise indicated, 250 μg sample lysates were added and incubated for either 4 h (Tat and DPPIV co-immunoprecipitation) or overnight (all other tests) at 4°C. Unbound proteins were removed and samples washed several times with washing buffer (PBS, pH 8.0 containing 0.5% n-octyl-β-D-glucopyranoside, 500 KU Trasylol and 0.5 mM DTT). The pellets containing the precipitates were subjected to western blot analyses.

### Western blot analysis

Immunoprecipitates were analyzed by SDS-PAGE under reducing and denaturing conditions. The samples were boiled for 5 min at 98°C in reducing sample buffer (250 mM Tris-HCl pH 6.8, 20% (v/v) glycerol, 0.01% bromophenol blue, 10% β-mercaptoethanol and 4% SDS) and separated by electrophoresis. Afterwards, the gels were blotted on nitrocellulose membrane at 250 mA for 1 h. The membranes were blocked with 5% non-fat milk in PBS, pH 8.0 containing 0.1% Tween 20 (Bio-Rad). Blocking for detection with anti-phospho-tyrosine mAb was performed with 3% BSA in TBS, pH 7.4. Probing for DPPIV and Tat protein was performed with anti-DPPIV pAb and anti-Tat mAb, respectively, with each antibody being diluted to 1: 1000 in PBS, pH 8.0 containing 0.1% Tween 20. Secondary antibodies were Dako-Envision horse-radish-peroxidase coupled anti-mouse/anti-rabbit IgG (Dako-North America Inc.) for mAb and pAb, respectively. To detect protein phosphorylated on serine- or tyrosine residues, anti-phospho-serine mAb (diluted to 1:1000 in PBS, pH 8.0 containing 0.1% Tween-20) and anti-phospho-tyrosine mAb (diluted to 1:5000 in TBS, pH 7.4 containing 1.5% BSA and 0.5% Tween 20) were used, respectively in combination with the Dako-Envision HRP-anti-mouse IgG.

### Quantification of protein band intensities and determination of relative phosphorylation levels

After western blotting, the intensities of phospho-Tat or phospho-DPPIV protein bands resulting from immunoprecipitation with either anti-phospho-serine mAb or anti-phospho-tyrosine mAb respectively, were quantified with the volume contour tool of the QuantityOne software (Bio-Rad) and calibrated against the protein band intensities of respective samples precipitated with anti-Tat mAb or anti-DPPIV pAb respectively. To deduce the relative phosphorylation fold of phospho-DPPIV, DPPIV expressed alone for 72 h without induction with purified Tat protein was set as standard (= 1). The quotients of the resulting values of respective phospho-DPPIV samples after calibration and that of the standard were then deduced as phosphorylation fold. The relative phosphorylation level of Tat protein was derived as follows: after calibration with total Tat protein, the level of phospho-Tat protein expressed alone for 72 h was considered as standard and taken as 100%. The levels of phospho-Tat protein in all the other test samples were then calculated as a percentage of this standard.

## List of abbreviations

GST: Glutathione-S-Transferase; HIV: human immunodeficiency virus; AIDS: acquired immune deficiency syndrome; GTH: GST-Tat-His; MALDI-MS: matrix-assisted laser-desorption ionisation mass spectrometry.

## Competing interests

The authors declare that they have no competing interests.

## Authors' contributions

FLT performed the experiments and wrote the draft manuscript. VB, MB and RT conducted the MALDI-MS measurements included as additional data and participated in the final review of the manuscript. HF coordinated the study, made substantial contributions to analysis and interpretation of the data and participated in the review of the manuscript together with WR. All authors read and approved the final manuscript.

## Supplementary Material

Additional file 1 **Negative controls for confocal microscopy**. *Sf9 *cells either infected with the Tat- or DPPIV-recombinant baculovirus were harvested after 72 h then fixed on culture slides and double-stained with the antibody combination: anti-Tat mAb/anti-mouse-Cy3 and anti-DPPIV pAb/anti-rabbit-FITC. Nuclei were stained with DAPI and images made at a 100-fold magnification by confocal laser scanning microscopy. Unspecific binding of the anti-Tat mAb to host proteins of DPPIV expressing cells (A) or the anti-DPPIV pAb to host proteins of Tat-expressing cells (B) did not take place. Furthermore, *Sf9 *cells co-infected with Tat and DPPIV recombinant baculoviruses were harvested after 72 h then fixed on culture slides and double-stained with the antibody combination: anti-Tat mAb/anti-mouse-Cy3/anti-rabbit-FITC (C), or with anti-DPPIV pAb/anti-rabbit-FITC/anti-mouse-Cy3 (D). Nuclei were stained with DAPI and images were made at a 100-fold magnification by confocal laser scanning microscopy under the same conditions like the test cells stained with both anti-Tat mAb and anti-DPPIV pAb. Click here for file

Additional file 2 **Determination of the transactivation activity of recombinant Tat protein**. The HIV-Tat protein binds to an RNA structure which leads to the activation of transcriptional initiation and elongation from the HIV-LTR promoter. The cell line HLCD4CAT (Centralised Facility for AIDS Reagents), stably transfected with the chloramphenicol acetyl transferase (CAT) reporter gene linked to the 5'-LTR of HIV1 was cultured on 6 well plates and grown for 24 h to achieve 60-80% confluency. Prior to treatment with the purified Tat protein the medium was aspirated and the cells washed once with 2 ml PBS, pH 7.2. The protein samples (controlled for endotoxin content) were diluted with PBS to a final concentration 0.1 μg/μl. Aliquots were mixed with culture medium to a final concentration 125 ng/ml then added to the cells. After culturing for 24 h the medium was changed against fresh culture medium and the cells subjected to a further incubation for 24 h. Purified non-tagged recombinant Tat protein (Immuno-Diagnostics, USA) was used as positive control. Tat storage buffer (50 mM Tris pH 8.0, 200 mM KCl, 5 mM DTT), the Tat translocation peptide Tat47-57 (Genscript Corp) and purified GST protein were used as negative controls. The cells were harvested and evaluated for the expression of CAT protein by use of the commercially available CAT-ELISA kit (Pierce, Rockford, USA). Briefly, cells were lysed and fractionated by centrifugation. The total protein concentration of each sample was determined by the BCA method and equivalent protein concentrations per sample were used for CAT assay according to manufacturer's instructions. The amount of CAT expressed/ml/μg Tat protein used was determined and the results of 3 independent experiments ± standard deviation presented in a bar diagram. The purified GST-Tat-His protein revealed the highest transactivation capacity. The Tat protein from Immuno-Diagnostics (Tat +ve) which served as a positive control also revealed high transactivation ability. Compared to this, the commercial Tat47-57 peptide was unable to activate the HIV-LTR, which is in accordance with the fact that recognition and binding of the HIV transacting response element on the LTR and activation requires other domains of the HIV-Tat protein. Click here for file

Additional file 3 **Determination of the ability of purified recombinant Tat protein to inhibit the cleavage of Glucagon-Like-Peptide-1 by purified human-DPPIV protein**. DPPIV cleavage of Glucagon-like-peptide 1 (GLP1, Genscript Corp) was evaluated by measuring the mass spectra of cleaved substrate at different time points by MALDI-TOF mass spectrometry. To proof the inhibitory effect of the HIV1-Tat protein on the enzymatic activity of DPPIV, *E. coli *expressed, full length Tat-1-86 protein (Immuno-Diagnostics, USA) and the GST-Tat-His protein bearing an N-terminal GST-fusion tag were used. The human-DPPIV protein used was expressed in *Sf9 *cells and purified in two-steps by immuno-affinity chromatography and size-exclusion chromatography. A 100 μl assay sample composed of (final concentrat ions) 17 mM Tris pH 8.0, 20 mM KCl, 15 mM NaCl, 0.5 mM DTT, 16 nM DPPIV, 31.25 μM GLP1 and either 1 μM Tat-1-86 or 1 μM GST-Tat-His. The substrate was added last in each test. For assays without Tat, equivalent volume of Tat storage buffer (50 mM Tris pH 8.0, 200 mM KCl, 5 mM DTT) was added to the mixture. After pippetting all assay components, 5 μl was qui ckly removed and added to 0.5 μl of a 1% tri-fluoro acetic acid (TFA) solution to stop the reaction. This sample was at time, t = 0. The assay mixture was incubated at 37°C and aliquots of 5 μl removed at 5 min intervals (for 15 min) and stopped with TFA. 1 μl of the re action mixtures at time-points t = 0, 5, 10 and 15 min were spotted on a MALDI target and the masses of proteolytically derived GLP1 were measured on a Bruker Ultra Flex-III MALDI-TOF mass spectrometer (Bruker, Bremen, Germany) with α-cyano-4-hydroxycinnamic acid (ACCA, 10 mg/ml in 70% acetonitrile and 0.1% TFA) as matrix. The mass spectra of GLP1 were monitored during cleavage by DPPIV in the absence of Tat protein (A) or in the presence of 1 μM Tat (B) or 1 μM GST-Tat-His (C) respectively. Uncleaved GLP1(7-36) has a mass of 3296 Da, whereas GLP1(9-36) results from proteolytic cleavage by DPPIV and has a mass of about 3088 Da. In the absence of Tat protein and in the presence of the GST-Tat-His protein, 16 nM DPPIV cleaved up 31.25 μM GLP1 within 5 min, indicating that the GST-Tat-His has no inhibitory effect on DPPIV. In the presence of 1 μM of the Tat-1-86 only about 40% of the GLP1 was cleaved after 5 min, indicating that Tat protein with the free N-terminus has inhibitory effect on the enzymatic activity of DPPIV. Click here for file

## References

[B1] Hegen M Niedobitek G Klein CE Stein H Fleischer B The T cell triggering molecule Tp103 is associated with dipeptidyl aminopeptidase IV activity J Immunol 1990 144 2908 2914 1969875

[B2] Misumi Y Hayashi Y Arakawa F Ikehara Y Molecular cloning and sequence analysis of human dipeptidyl peptidase IV, a serine proteinase on the cell surface Biochim Biophys Acta 1992 1131 333 336 1352704 10.1016/0167-4781(92)90036-y

[B3] Callahan PX McDonald JK Ellis S Dipeptidyl aminopeptidase I: application in sequencing of peptides Fed Proc 1972 31 1105 1113 4338109

[B4] Kenny AJ Booth AG Wood EJ Young AR Dipeptidyl peptidase IV, a kidney microvillus serine proteinase: evidence for its large subunit molecular weight and endopeptidase activity Biochem Soc Trans 1976 4 347 348 1001687 10.1042/bst0040347

[B5] Hanski C Huhle T Reutter W Involvement of plasma membrane dipeptidyl peptidase IV in fibronectin-mediated adhesion of cells on collagen Biol Chem Hoppe Seyler 1985 366 1169 1176 2868739 10.1515/bchm3.1985.366.2.1169

[B6] Löster K Zeilinger K Schuppan D Reutter W The cysteine-rich region of dipeptidyl peptidase IV (CD 26) is the collagen-binding site Biochem Biophys Res Commun 1995 217 341 348 10.1006/bbrc.1995.2782 8526932

[B7] Kameoka J Tanaka T Nojima Y Schlossman SF Morimoto C Direct association of adenosine deaminase with a T cell activation antigen, CD26 Science 1993 261 466 469 10.1126/science.8101391 8101391

[B8] De Meester I Vanham G Kestens L Vanhoof G Bosmans E Gigase P Scharpe S Binding of adenosine deaminase to the lymphocyte surface via CD26 Eur J Immunol 1994 24 566 570 10.1002/eji.1830240311 7907293

[B9] Morimoto C Schlossman SF The structure and function of CD26 in the T-cell immune response Immunol Rev 1998 161 55 70 10.1111/j.1600-065X.1998.tb01571.x 9553764

[B10] Broder CC Nussbaum O Gutheil WG Bachovchin WW Berger EA CD26 antigen and HIV fusion? Science 1994 264 1156 1159 author reply 1162-1155 10.1126/science.7909959 7909959

[B11] Callebaut C Krust B Jacotot E Hovanessian AG T cell activation antigen, CD26, as a cofactor for entry of HIV in CD4+ cells Science 1993 262 2045 2050 10.1126/science.7903479 7903479

[B12] Struyf S Proost P Sozzani S Mantovani A Wuyts A De Clercq E Schols D Van Damme J Enhanced anti-HIV-1 activity and altered chemotactic potency of NH2-terminally processed macrophage-derived chemokine (MDC) imply an additional MDC receptor J Immunol 1998 161 2672 2675 9743322

[B13] Mantovani A Gray PA Van Damme J Sozzani S Macrophage-derived chemokine (MDC) J Leukoc Biol 2000 68 400 404 10985257

[B14] Oravecz T Pall M Roderiquez G Gorrell MD Ditto M Nguyen NY Boykins R Unsworth E Norcross MA Regulation of the receptor specificity and function of the chemokine RANTES (regulated on activation, normal T cell expressed and secreted) by dipeptidyl peptidase IV (CD26)-mediated cleavage J Exp Med 1997 186 1865 1872 10.1084/jem.186.11.1865 9382885 PMC2199148

[B15] Appay V Rowland-Jones SL RANTES: a versatile and controversial chemokine Trends Immunol 2001 22 83 87 10.1016/S1471-4906(00)01812-3 11286708

[B16] Shioda T Kato H Ohnishi Y Tashiro K Ikegawa M Nakayama EE Hu H Kato A Sakai Y Liu H Honjo T Nomoto A Iwamoto A Morimoto C Nagai Y Anti-HIV-1 and chemotactic activities of human stromal cell-derived factor 1alpha (SDF-1alpha) and SDF-1beta are abolished by CD26/dipeptidyl peptidase IV-mediated cleavage Proc Natl Acad Sci USA 1998 95 6331 6336 10.1073/pnas.95.11.6331 9600965 PMC27682

[B17] Herrera C Morimoto C Blanco J Mallol J Arenzana F Lluis C Franco R Comodulation of CXCR4 and CD26 in human lymphocytes J Biol Chem 2001 276 19532 19539 10.1074/jbc.M004586200 11278278

[B18] Gutheil WG Subramanyam M Flentke GR Sanford DG Munoz E Huber BT Bachovchin WW Human immunodeficiency virus 1 Tat binds to dipeptidyl aminopeptidase IV (CD26): a possible mechanism for Tat's immunosuppressive activity Proc Natl Acad Sci USA 1994 91 6594 6598 10.1073/pnas.91.14.6594 7912830 PMC44249

[B19] Smith RE Talhouk JW Brown EE Edgar SE The significance of hypersialylation of dipeptidyl peptidase IV (CD26) in the inhibition of its activity by Tat and other cationic peptides. CD26: a subverted adhesion molecule for HIV peptide binding AIDS Res Hum Retroviruses 1998 14 851 868 10.1089/aid.1998.14.851 9671214

[B20] Ohtsuki T Tsuda H Morimoto C Good or evil: CD26 and HIV infection J Dermatol Sci 2000 22 152 160 10.1016/S0923-1811(99)00081-X 10698152

[B21] Weihofen WA Liu J Reutter W Saenger W Fan H Crystal structures of HIV-1 Tat-derived nonapeptides Tat-(1-9) and Trp2-Tat-(1-9) bound to the active site of dipeptidyl-peptidase IV (CD26) J Biol Chem 2005 280 14911 14917 10.1074/jbc.M413400200 15695814

[B22] Xiao H Neuveut C Tiffany HL Benkirane M Rich EA Murphy PM Jeang KT Selective CXCR4 antagonism by Tat: implications for in vivo expansion of coreceptor use by HIV-1 Proc Natl Acad Sci USA 2000 97 11466 11471 10.1073/pnas.97.21.11466 11027346 PMC17223

[B23] Jeang KT Chang Y Berkhout B Hammarskjold ML Rekosh D Regulation of HIV expression: mechanisms of action of Tat and Rev AIDS 1991 5 Suppl 2 S3 14 10.1097/00002030-199101001-00002 1845057

[B24] Dingwall C Ernberg I Gait MJ Green SM Heaphy S Karn J Lowe AD Singh M Skinner MA HIV-1 tat protein stimulates transcription by binding to a U-rich bulge in the stem of the TAR RNA structure EMBO J 1990 9 4145 4153 2249668 10.1002/j.1460-2075.1990.tb07637.x PMC552188

[B25] Karn J Tackling Tat J Mol Biol 1999 293 235 254 10.1006/jmbi.1999.3060 10550206

[B26] Gatignol A Jeang KT Tat as a transcriptional activator and a potential therapeutic target for HIV-1 Adv Pharmacol 2000 48 209 227 full_text 10987092 10.1016/s1054-3589(00)48007-5

[B27] Huigen MC Kamp W Nottet HS Multiple effects of HIV-1 trans-activator protein on the pathogenesis of HIV-1 infection Eur J Clin Invest 2004 34 57 66 10.1111/j.1365-2362.2004.01282.x 14984439

[B28] Vendeville A Rayne F Bonhoure A Bettache N Montcourrier P Beaumelle B HIV-1 Tat enters T cells using coated pits before translocating from acidified endosomes and eliciting biological responses Mol Biol Cell 2004 15 2347 2360 10.1091/mbc.E03-12-0921 15020715 PMC404028

[B29] Tyagi M Rusnati M Presta M Giacca M Internalization of HIV-1 tat requires cell surface heparan sulfate proteoglycans J Biol Chem 2001 276 3254 3261 10.1074/jbc.M006701200 11024024

[B30] Barillari G Sgadari C Fiorelli V Samaniego F Colombini S Manzari V Modesti A Nair BC Cafaro A Sturzl M Ensoli B The Tat protein of human immunodeficiency virus type-1 promotes vascular cell growth and locomotion by engaging the alpha5beta1 and alphavbeta3 integrins and by mobilizing sequestered basic fibroblast growth factor Blood 1999 94 663 672 10397733

[B31] Ruoslahti E Pierschbacher MD New perspectives in cell adhesion: RGD and integrins Science 1987 238 491 497 10.1126/science.2821619 2821619

[B32] Westendorp MO Li-Weber M Frank RW Krammer PH Human immunodeficiency virus type 1 Tat upregulates interleukin-2 secretion in activated T cells J Virol 1994 68 4177 4185 8207793 10.1128/jvi.68.7.4177-4185.1994 PMC236340

[B33] Buonaguro L Buonaguro FM Giraldo G Ensoli B The human immunodeficiency virus type 1 Tat protein transactivates tumor necrosis factor beta gene expression through a TAR-like structure J Virol 1994 68 2677 2682 8139045 10.1128/jvi.68.4.2677-2682.1994 PMC236745

[B34] Huang L Bosch I Hofmann W Sodroski J Pardee AB Tat protein induces human immunodeficiency virus type 1 (HIV-1) coreceptors and promotes infection with both macrophage-tropic and T-lymphotropic HIV-1 strains J Virol 1998 72 8952 8960 9765440 10.1128/jvi.72.11.8952-8960.1998 PMC110312

[B35] Howcroft TK Strebel K Martin MA Singer DS Repression of MHC class I gene promoter activity by two-exon Tat of HIV Science 1993 260 1320 1322 10.1126/science.8493575 8493575

[B36] Verhoef K Bauer M Meyerhans A Berkhout B On the role of the second coding exon of the HIV-1 Tat protein in virus replication and MHC class I downregulation AIDS Res Hum Retroviruses 1998 14 1553 1559 10.1089/aid.1998.14.1553 9840288

[B37] Kiernan RE Vanhulle C Schiltz L Adam E Xiao H Maudoux F Calomme C Burny A Nakatani Y Jeang KT Benkirane M Van Lint C HIV-1 tat transcriptional activity is regulated by acetylation EMBO J 1999 18 6106 6118 10.1093/emboj/18.21.6106 10545121 PMC1171675

[B38] Ammosova T Berro R Jerebtsova M Jackson A Charles S Klase Z Southerland W Gordeuk VR Kashanchi F Nekhai S Phosphorylation of HIV-1 Tat by CDK2 in HIV-1 transcription Retrovirology 2006 3 78 10.1186/1742-4690-3-78 17083724 PMC1636661

[B39] Van Duyne R Easley R Wu W Berro R Pedati C Klase Z Kehn-Hall K Flynn EK Symer DE Kashanchi F Lysine methylation of HIV-1 Tat regulates transcriptional activity of the viral LTR Retrovirology 2008 5 40 10.1186/1742-4690-5-40 18498648 PMC2412914

[B40] Blanco J Marie I Callebaut C Jacotot E Krust B Hovanessian AG Specific binding of adenosine deaminase but not HIV-1 transactivator protein Tat to human CD26 Exp cell Res 1996 225 102 111 10.1006/excr.1996.0161 8635502

[B41] Wrenger S Hoffmann T Faust J Mrestani-Klaus C Brandt W Neubert K Kraft M Olek S Frank R Ansorge S Reinhold D The N-terminal structure of HIV-1 Tat is required for suppression of CD26-dependent T cell growth J Biol Chem 1997 272 30283 30288 10.1074/jbc.272.48.30283 9374514

[B42] Boulanger MC Liang C Russell RS Lin R Bedford MT Wainberg MA Richard S Methylation of Tat by PRMT6 regulates human immunodeficiency virus type 1 gene expression J Virol 2005 79 124 131 10.1128/JVI.79.1.124-131.2005 15596808 PMC538702

[B43] Bres V Kiernan RE Linares LK Chable-Bessia C Plechakova O Treand C Emiliani S Peloponese JM Jeang KT Coux O Scheffner M Benkirane M A non-proteolytic role for ubiquitin in Tat-mediated transactivation of the HIV-1 promoter Nat Cell Biol 2003 5 754 761 10.1038/ncb1023 12883554

[B44] Wrenger S Reinhold D Hoffmann T Kraft M Frank R Faust J Neubert K Ansorge S The N-terminal X-X-Pro sequence of the HIV-1 Tat protein is important for the inhibition of dipeptidyl peptidase IV (DP IV/CD26) and the suppression of mitogen-induced proliferation of human T cells FEBS Lett 1996 383 145 149 10.1016/0014-5793(96)00221-9 8925885

[B45] Borgatti PZG Colamussi ML Gibellini D Previati M Cantley LL Capitani S Extracellular HIV-1 Tat protein activates phosphatidylinositol 3- and Akt/PKB kinases in CD4+ T lymphoblastoid Jurkat cells Eur J Immunol 1997 11 2805 2811 10.1002/eji.1830271110 9394803

[B46] Gibellini DBA Pierpaoli S Bertolaso L Milani D Capitani S La Placa M Zauli G Extracellular HIV-1 Tat protein induces the rapid Ser133 phosphorylation and activation of CREB transcription factor in both Jurkat lymphoblastoid T cells and primary peripheral blood mononuclear cells J Immunol 1998 160 3891 3898 9558095

[B47] Menegon ALC Benfenati F Valtorta F Tat protein from HIV-1 activates MAP kinase in granular neurons and glial cells from rat cerebellum Biochem Biophys Res Commun 1997 238 800 805 10.1006/bbrc.1997.7393 9325171

[B48] Kahne T Neubert K Faust J Ansorge S Early phosphorylation events induced by DPIV/CD26-specific inhibitors Cell Immunol 1998 189 60 66 10.1006/cimm.1998.1355 9758695

[B49] Fox DA Hussey RE Fitzgerald KA Acuto O Poole C Palley L Daley JF Schlossman SF Reinherz EL Ta1, a novel 105 KD human T cell activation antigen defined by a monoclonal antibody J Immunol 1984 133 1250 1256 6205075

[B50] Li CJ Friedman DJ Wang C Metelev V Pardee AB Induction of apoptosis in uninfected lymphocytes by HIV-1 Tat protein Science 1995 268 429 431 10.1126/science.7716549 7716549

[B51] Richter B Bandeira-Echtler E Bergerhoff K Lerch C Emerging role of dipeptidyl peptidase-4 inhibitors in the management of type 2 diabetes Vasc Health Risk Manag 2008 4 753 768 19065993 10.2147/vhrm.s1707 PMC2597770

[B52] Bilodeau N Fiset A Poirier GG Fortier S Gingras MC Lavoie JN Faure RL Insulin-dependent phosphorylation of DPP IV in liver. Evidence for a role of compartmentalized c-Src FEBS J 2006 273 992 1003 10.1111/j.1742-4658.2006.05125.x 16478473

[B53] Aertgeerts K Ye S Tennant MG Kraus ML Rogers J Sang BC Skene RJ Webb DR Prasad GS Crystal structure of human dipeptidyl peptidase IV in complex with a decapeptide reveals details on substrate specificity and tetrahedral intermediate formation Protein Sci 2004 13 412 421 10.1110/ps.03460604 14718659 PMC2286704

[B54] Tahirov TH Babayeva ND Varzavand K Cooper JJ Sedore SC Price DH Crystal structure of HIV-1 Tat complexed with human P-TEFb Nature 2010 465 747 751 10.1038/nature09131 20535204 PMC2885016

[B55] Dobers J Zimmermann-Kordmann M Leddermann M Schewe T Reutter W Fan H Expression, purification, and characterization of human dipeptidyl peptidase IV/CD26 in *Sf9 *insect cells Protein Expr Purif 2002 25 527 532 10.1016/S1046-5928(02)00043-8 12182835

[B56] Sanger F Nicklen S Coulson AR DNA sequencing with chain-terminating inhibitors Proc Natl Acad Sci USA 1977 74 5463 5467 10.1073/pnas.74.12.5463 271968 PMC431765

