# Supplementary figures and images for: Interaction of human dipeptidyl peptidase IV and human immunodeficiency virus type-1 transcription transactivator in Sf9 cells
Source: Virol J. 2010 Oct 13;7:267. doi: 10.1186/1743-422X-7-267 (PMC2967539; doi:10.1186/1743-422X-7-267)

Negative controls for confocal microscopy

A

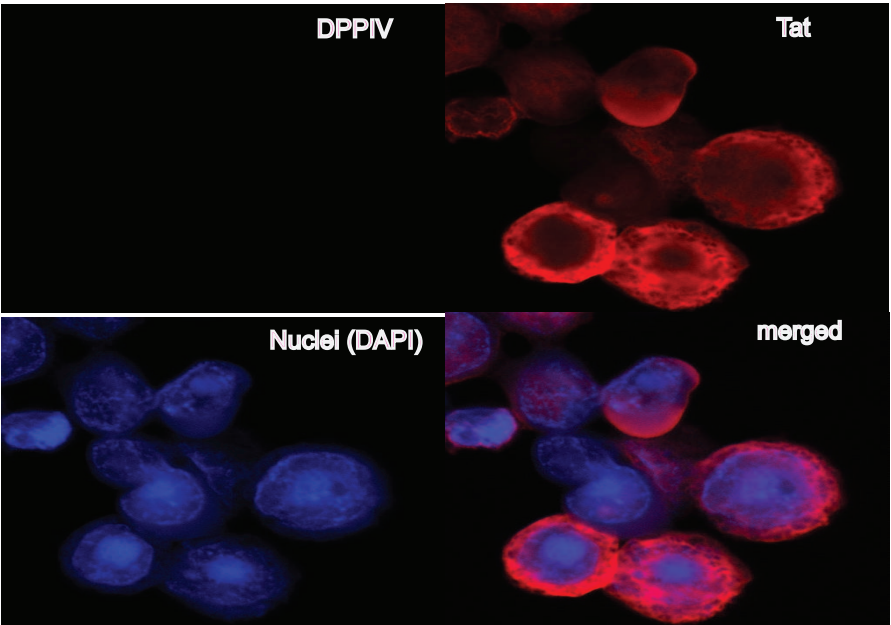

C

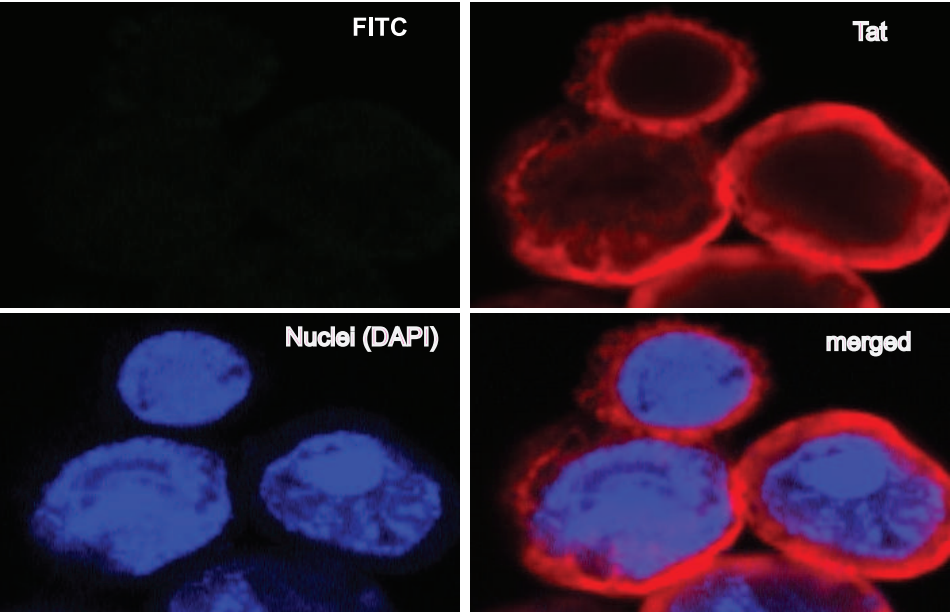

B

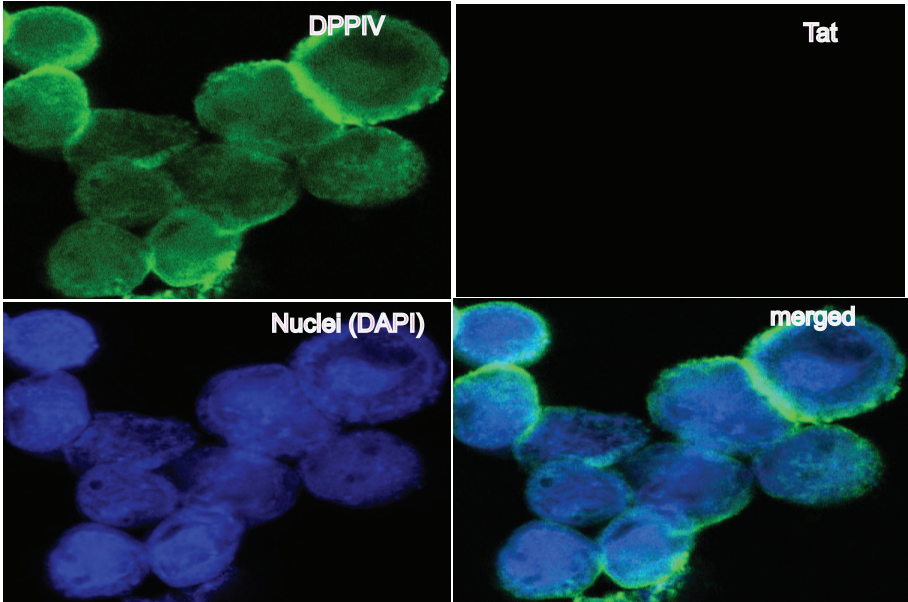

D

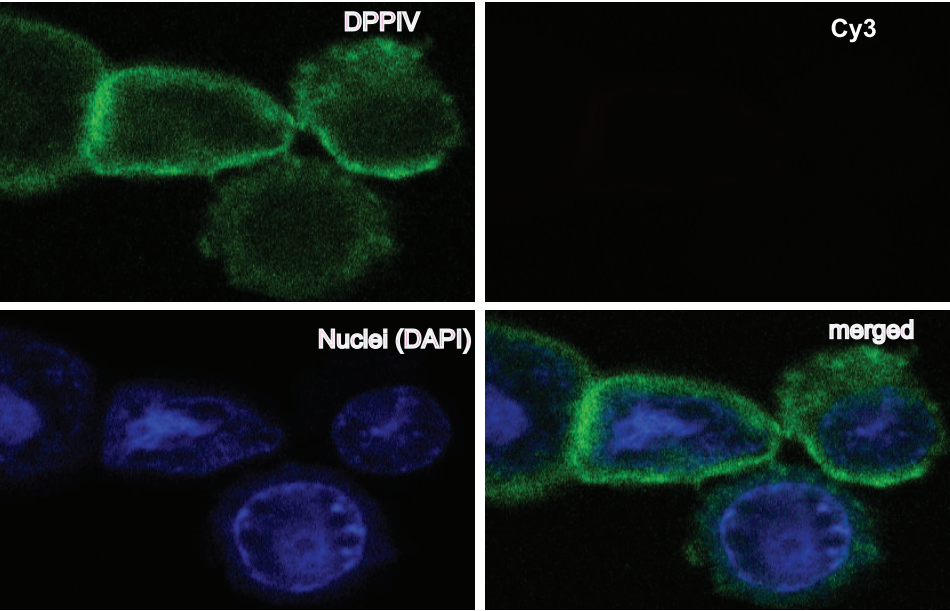

Supplement: Additional file 1 — Negative controls for confocal microscopy. Sf9 cells either infected with the Tat- or DPPIV-recombinant baculovirus were harvested after 72 h then fixed on culture slides and double-stained with the antibody combination: anti-Tat mAb/anti-mouse-Cy3 and anti-DPPIV pAb/anti-rabbit-FITC. Nuclei were stained with DAPI and images made at a 100-fold magnification by confocal laser scanning microscopy. Unspecific binding of the anti-Tat mAb to host proteins of DPPIV expressing cells (A) or the anti-DPPIV pAb to host proteins of Tat-expressing cells (B) did not take place. Furthermore, Sf9 cells co-infected with Tat and DPPIV recombinant baculoviruses were harvested after 72 h then fixed on culture slides and double-stained with the antibody combination: anti-Tat mAb/anti-mouse-Cy3/anti-rabbit-FITC (C), or with anti-DPPIV pAb/anti-rabbit-FITC/anti-mouse-Cy3 (D). Nuclei were stained with DAPI and images were made at a 100-fold magnification by confocal laser scanning microscopy under the same conditions like the test cells stained with both anti-Tat mAb and anti-DPPIV pAb. [file 1743-422X-7-267-S1.PDF]

Determination of the transactivation activity of recombinant Tat protein

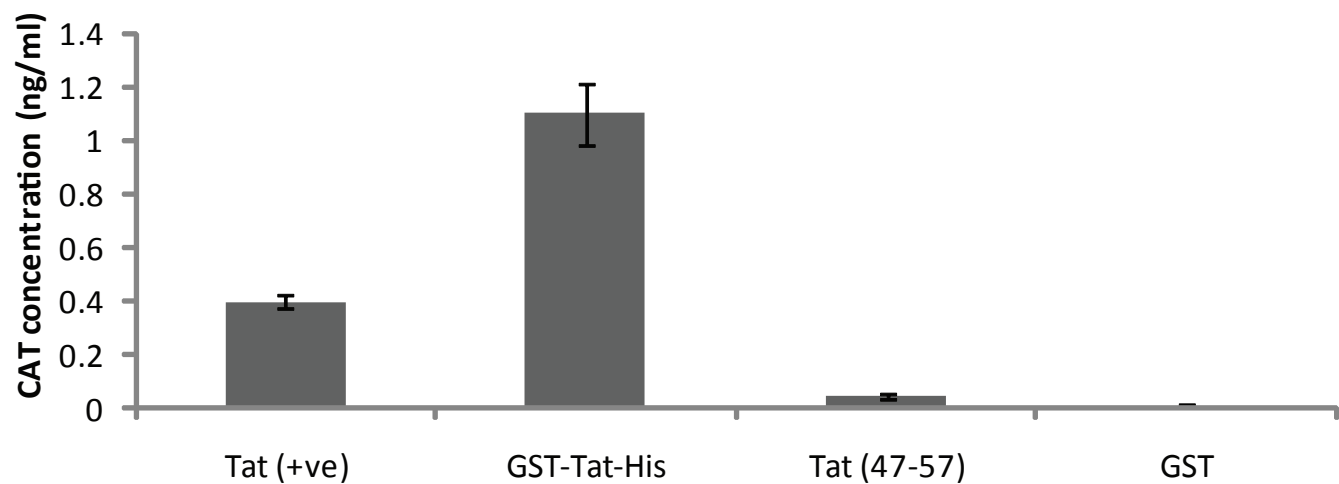

Supplement: Additional file 2 — Determination of the transactivation activity of recombinant Tat protein. The HIV-Tat protein binds to an RNA structure which leads to the activation of transcriptional initiation and elongation from the HIV-LTR promoter. The cell line HLCD4CAT (Centralised Facility for AIDS Reagents), stably transfected with the chloramphenicol acetyl transferase (CAT) reporter gene linked to the 5'-LTR of HIV1 was cultured on 6 well plates and grown for 24 h to achieve 60-80% confluency. Prior to treatment with the purified Tat protein the medium was aspirated and the cells washed once with 2 ml PBS, pH 7.2. The protein samples (controlled for endotoxin content) were diluted with PBS to a final concentration 0.1 μg/μl. Aliquots were mixed with culture medium to a final concentration 125 ng/ml then added to the cells. After culturing for 24 h the medium was changed against fresh culture medium and the cells subjected to a further incubation for 24 h. Purified non-tagged recombinant Tat protein (Immuno-Diagnostics, USA) was used as positive control. Tat storage buffer (50 mM Tris pH 8.0, 200 mM KCl, 5 mM DTT), the Tat translocation peptide Tat47-57 (Genscript Corp) and purified GST protein were used as negative controls. The cells were harvested and evaluated for the expression of CAT protein by use of the commercially available CAT-ELISA kit (Pierce, Rockford, USA). Briefly, cells were lysed and fractionated by centrifugation. The total protein concentration of each sample was determined by the BCA method and equivalent protein concentrations per sample were used for CAT assay according to manufacturer's instructions. The amount of CAT expressed/ml/μg Tat protein used was determined and the results of 3 independent experiments ± standard deviation presented in a bar diagram. The purified GST-Tat-His protein revealed the highest transactivation capacity. The Tat protein from Immuno-Diagnostics (Tat +ve) which served as a positive control also revealed high transactivation ability. C [file 1743-422X-7-267-S2.PDF]
