# Supplementary material for: Interaction of human dipeptidyl peptidase IV and human immunodeficiency virus type-1 transcription transactivator in Sf9 cells
Source: Virol J. 2010 Oct 13;7:267. doi: 10.1186/1743-422X-7-267 (PMC2967539; doi:10.1186/1743-422X-7-267)

Determination of the ability of purified recombinant Tat protein to inhibit the cleavage of Glucagon-Like-Peptide-1 by purified human-DPPIV protein

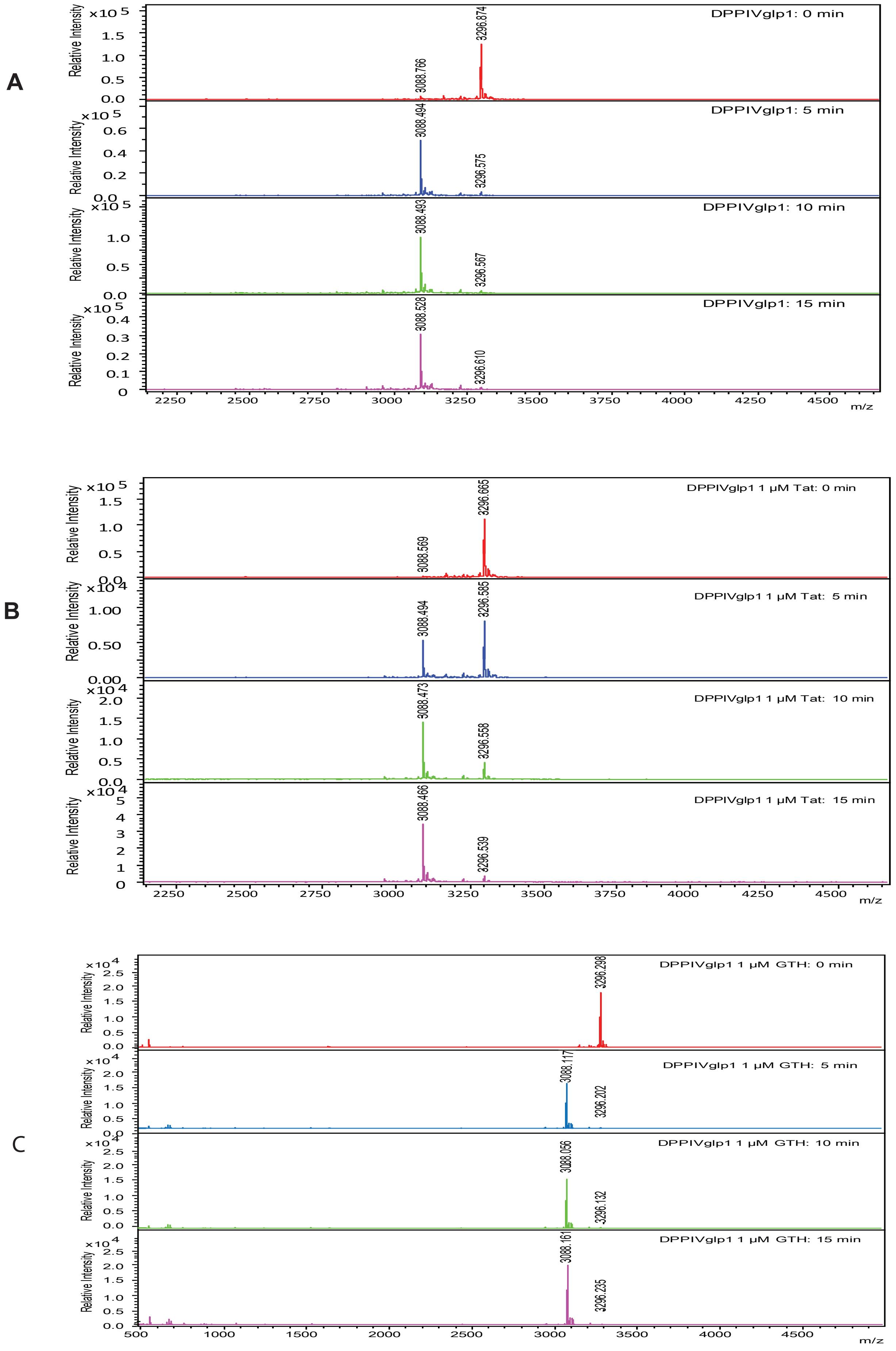

Supplement: Additional file 3 — Determination of the ability of purified recombinant Tat protein to inhibit the cleavage of Glucagon-Like-Peptide-1 by purified human-DPPIV protein. DPPIV cleavage of Glucagon-like-peptide 1 (GLP1, Genscript Corp) was evaluated by measuring the mass spectra of cleaved substrate at different time points by MALDI-TOF mass spectrometry. To proof the inhibitory effect of the HIV1-Tat protein on the enzymatic activity of DPPIV, E. coli expressed, full length Tat-1-86 protein (Immuno-Diagnostics, USA) and the GST-Tat-His protein bearing an N-terminal GST-fusion tag were used. The human-DPPIV protein used was expressed in Sf9 cells and purified in two-steps by immuno-affinity chromatography and size-exclusion chromatography. A 100 μl assay sample composed of (final concentrat ions) 17 mM Tris pH 8.0, 20 mM KCl, 15 mM NaCl, 0.5 mM DTT, 16 nM DPPIV, 31.25 μM GLP1 and either 1 μM Tat-1-86 or 1 μM GST-Tat-His. The substrate was added last in each test. For assays without Tat, equivalent volume of Tat storage buffer (50 mM Tris pH 8.0, 200 mM KCl, 5 mM DTT) was added to the mixture. After pippetting all assay components, 5 μl was qui ckly removed and added to 0.5 μl of a 1% tri-fluoro acetic acid (TFA) solution to stop the reaction. This sample was at time, t = 0. The assay mixture was incubated at 37°C and aliquots of 5 μl removed at 5 min intervals (for 15 min) and stopped with TFA. 1 μl of the re action mixtures at time-points t = 0, 5, 10 and 15 min were spotted on a MALDI target and the masses of proteolytically derived GLP1 were measured on a Bruker Ultra Flex-III MALDI-TOF mass spectrometer (Bruker, Bremen, Germany) with α-cyano-4-hydroxycinnamic acid (ACCA, 10 mg/ml in 70% acetonitrile and 0.1% TFA) as matrix. The mass spectra of GLP1 were monitored during cleavage by DPPIV in the absence of Tat protein (A) or in the presence of 1 μM Tat (B) or 1 μM GST-Tat-His (C) respectively. Uncleaved GLP1(7-36) has a mass of 3296 Da, whereas GLP1(9-36) results from proteolytic clea [file 1743-422X-7-267-S3.PDF]
